# Infant oral mutilation (IOM) related to traditional practices among inner city pre-school children in Sudan

**DOI:** 10.4314/ahs.v18i2.21

**Published:** 2018-06

**Authors:** Alya Isam Elgamri, Azza Tagelsir Ahmed, Omer Elfatih Haj-Siddig, Judith R Chin

**Affiliations:** 1 Department of Paediatric Dentistry, University of Khartoum, Sudan; 2 Department of Cariology, Operative Dentistry, and Dental Public Health, Indiana University School of Dentistry, USA; 3 Ministry of Health, Sudan; 4 Department of Pediatric Dentistry, Nova Southeastern University, USA

**Keywords:** Infant oral mutilation (IOM), traditional practices, pre-school children, Sudan

## Abstract

**Background:**

The term Infant Oral Mutilation (IOM) refers to the aggressive cultural rituals where primary canine tooth germs of infants are enucleated for therapeutic reasons.

**Objectives:**

To determine the prevalence and risk factors for IOM among inner city pre-school children in Khartoum.

**Methods:**

In this cross-sectional study, 212 randomly selected children from twelve government pre-schools in Khartoum were examined for the presence of IOM. Socio-demographic, feeding and teething-related data were collected by self-administered questionnaires.

**Results:**

The mean age of the sample was 4.7 years. The prevalence of clinical IOM was 10.8%. Multivariable regression analysis revealed that children who suffered from diarrhea during teething were 7.15 times more likely to have clinical IOM over their counterparts (p<0.0001). Mothers who were educated below elementary school level were 2.69 times more likely to have children showing clinical IOM (p= 0.0369).

**Conclusion:**

The present study showed that the practice of IOM is common among inner city children. Certain teething-related symptoms especially diarrhea and maternal education could be strong determinants of the malpractice of IOM.

## Introduction

Infant oral mutilation (IOM) is the term used to describe the traditional practice in which the primary canine tooth germs are enucleated in an attempt to alleviate teething - associated symptoms such as diarrhea, vomiting and fever in infants^1^. This East African practice is well described in the literature under different terminologies such as “Meno ya plastiki”, “Lugbara tooth” extraction, “canine killer” extraction, Ibino/Ebino and germectomy^2^–^6^. In Sudan, the earliest published report of IOM practice came out from the southern parts of the Sudan by the late 80's^7^. While the Southern Sudanese adopted the terminology “Lugbara tooth” to describe the practice of extracting the unerupted primary canine follicle, referring to Lugbara Ugandan tribes^8^, the Northern Sudanese, adopted a less aggressive practice-termed “Haifat”, where the swollen alveolar process over the unerupted primary canines is lanced with a sharp, heated instrument until bleeding occurs^9^. There are inconsistent prevalence figures of IOM in the literature, with extremely high rates reported in Kenya^5^, Tanzania^6^ and South West Ethiopia^3^. However, a declining trend of IOM prevalence in some East African countries was assumed to be related to a shift in the practice itself as traditional healers changed their practice from surgical to non-surgical intervention such as the rubbing of herbs on the area^6^. African immigrants represent a small but a fast-growing group in industrialized western populations. The number of USA foreign-born immigrants from Africa has grown rapidly, roughly doubling each decade since the 1970^10^. With the significant escalating figures of international migration across the globe, the practice of IOM has gained much attention globally in recent years. Multiple case series and case reports from some European countries^11^–^14^, USA^15^ and Australia^16^ have documented the existence of IOM practices among immigrants of African origin. Some reports were able to confirm that the procedure of canine enucleation took place in the new destination countries^17^, others suggested that the procedures might have taken place during trips to the motherland^18^. IOM prevalence among African immigrant populations in recent reports has ranged from 20.8% in Sweden to as high as 60% in Israel^11^,^17^.

Apart from the psychological impact of such a detrimental ritual, the procedure of tooth bud enucleation has many short and long-term health-related repercussions. Short terms complications include pain, bleeding and septicaemia. The late consequences of this practice includes: failure of development of succedaneous teeth, enamel defects of the adjacent primary and permanent teeth, and development of peg-shaped permanent teeth^19^. Limited data is available from Northern Sudan. The only prevalence report has been published more than two decades ago and documented a prevalence of 22% among children aged 4–8 years^9^.

As there is a lack of updated prevalence data from Sudan, there appears to be an imperative need to explore the practice of IOM, particularly where this practice is rife. Therefore, the study primary objective was to determine the prevalence and socio-demographic and teething-related risk factors for IOM among inner city pre-school children in Khartoum.

## Methods

### Study setting

There are 17 federal states in Sudan, each of which is divided into localities (districts). Khartoum locality (district) is one of seven administrative units in Khartoum state and it incorporates Khartoum city, the national capital of Sudan. This cross-sectional study included a sample of children attending government pre-schools in Khartoum locality (district) in Khartoum state, Sudan. The total number of pre-school children in government kindergartens was 661 children distributed across 12 government pre-schools in Khartoum locality (official report as of November 2013).

### Sample size and sampling strategy

The sampling frame constituted all the governmental preschools (n=12) in Khartoum locality, with each preschool representing a stratum. For the sample calculation, we assumed that the expected proportion of IOM among children as equal to 22%^9^ and the marginal error as 5% of the expected proportion (z_α equal to 1.96 for 95% confidence level). Therefore for a total population size of 661 children attending these governmental preschools, the minimum required sample size was estimated to be around 190 children. Using a random starting point, every second child on the school list of the participating schools was given a consent form to be signed by parents/guardian. Total potential participants invited was 227 subjects. Children for whom parental consent could not be obtained and those who were not present on the day of the clinical examination were excluded, resulting in a final sample of 212 children.

### Calibration, Validity, and reproducibility

Two examiners (AE, OH) attended a short training session before the commencement of data collection. The training session included illustrations of the possible different clinical scenarios associated with the practice of IOM. Intra-examiner calibration was done by re-examining a subset of the of the sample (10%) and the Cohen's Kappa test was 0.82. For cases with doubtful diagnosis, a third investigator (AT) evaluated the case until a consensus was reached among the examiners.

### Data collection & Examination

Only children whose parents/guardians agreed to participate were included in the study. A clinical examination was performed and data was collected by means of self-administered questionnaires that were completed by the parents/ guardians. Most participants were able to complete the questionnaires but parents/guardians who found difficulty completing the questionnaires sought help from class teachers. The questionnaire was initially compiled in English and then translated into Arabic. Information regarding child demographics was recorded. This included age, gender, place of birth, residence geographical location in the district and educational background of parents. The subjects' status as city residents was obtained by asking questions related to the child place of birth and if they ever lived outside Khartoum for the first two years of their lives. Information regarding the child's feeding history included the type of feeding during infancy (exclusive breastfeeding, no breastfeeding/exclusive bottle feeding, or combination of both), and duration of breastfeeding (less than 6 months, 6–12 months, more than 12 months). The questionnaire also inquired if the child experienced teething problems (yes, no, I don't know). This section further included dichotomous questions about each possible teething symptom (fever, gum pain, drooling, diarrhoea, biting, vomiting, and others). The parents' approach to relieve teething problems was investigated i.e. “taking the child to doctor”, “giving medications only”, “taking the child to the traditional healer”, or “doing nothing”.

**Image 1:**
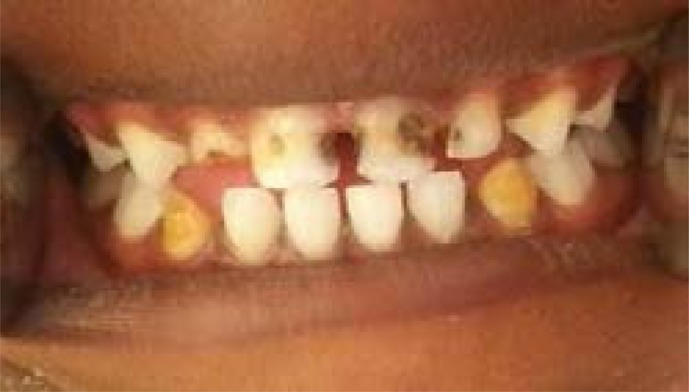
4 years old male, Enamel defect tooth #83& #73

**Image 2:**
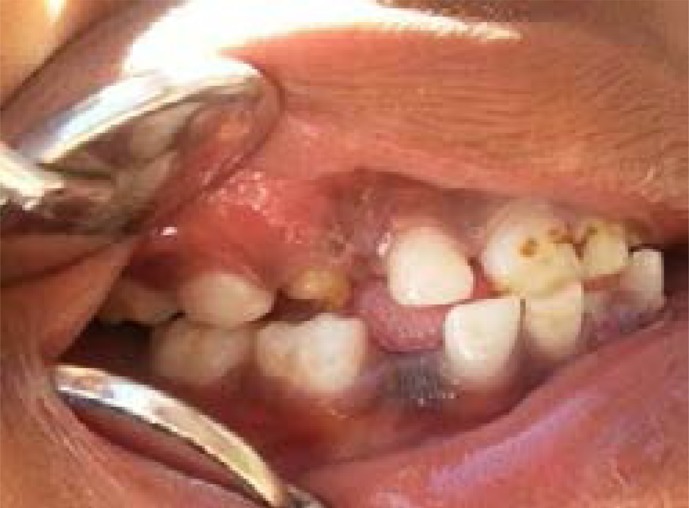
6 years old female, Missing tooth #83 and Enamel defect tooth #53

Two calibrated dentists (AE, OH) performed the oral clinical examinations. Examinations took place under the following standard conditions: subjects were seated on regular chairs under natural light, teeth were cleaned if necessary using cotton gauze. The examination was performed using dental mirrors for examination and lip retraction. After the examination, a session of oral health education was performed for the whole school and pamphlets were distributed. Children who needed dental treatment were referred to the nearest government dental care facility. Clinical examination included the status of the primary maxillary and mandibular canines, primary maxillary and mandibular lateral incisors, and first primary molars using the criteria suggested by Rodd & Davidson^18^. IOM was only considered when one or more of the following conditions were positive: (1) Absence of the primary maxillary or mandibular canine in children younger than 9 years and 7 years respectively, when others primary incisors were still present, (2) Presence of developmental enamel defects involving the buccal or incisal tip of a primary canines which was inconsistent with any other generalized enamel defect, and (3) Presence of any localized developmental defect of the maxillary and mandibular primary lateral incisors, canines or first molars. The status of index teeth was recorded as intact, missing or presence of enamel defect/s. Teeth were considered intact when no clinical defect was evident. Teeth were considered missing when the primary canine was absent but primary incisors were still present in the mouth (where conventional extraction could be excluded). Enamel defects were divided into enamel hypoplasia (grooves, pits, or generalized lack of enamel), localized or diffuse enamel opacities (intact white, yellow, or brown defects), and combined lesions where any of the above descriptions presented together. Enamel defects less than 1–2 mm in size were not recorded (Images 1–2).

### Statistical analysis

Basic descriptive statistics (counts, percentages, mean with standard deviations) were carried out. McNemar's Test for marginal homogeneity of clinical IOM and history of IOM was conducted. The outcome measure was determined as the prevalence of clinical IOM. The variable “clinical IOM” was defined as the presence of at least one positive criterion of Rodd & Davidson^18^ of any of the 12 indexed teeth, whereas the variable “history of IOM” was defined as a positive history of a visit to a traditional healer (to relieve teething symptoms) as reported by the parent(s)/guardian(s).

Chi-square tests and Fisher's Exact tests were used to compare IOM prevalence estimates and other potential predictors of IOM (gender, different residence geographical locations, place of birth, feeding and teething -related symptoms, and parental education background levels). For predicting “clinical IOM”, a multivariable logistic regression model was constructed from variables with a significance of ≤ 0.30 in the bivariate tests. A stepwise selection process was used to find the best model containing only variables with a significance level of <0.05.

## Results

### Sample characteristics

Of the 227 potential subjects approached, 212 children agreed to participate in this study (response rate 93.8%) of whom 53.8% were males and 48.2% were females. Subjects' mean age was 4.7 years (±0.88). Table 1 illustrates the socio-demographic details of the sample population.

**Table 1:** Frequency distribution (n, %) according to the socio-demographics of the sample (n=212) and correlation with Clinical IOM

Variable		N (%)	Clinical IOM	p-value*
**Gender**	Female	98 (46.2%)	8 (3.8%)	0.2437
	Male	114 (53.8%)	15 (7.1%)	
**Residence geographical** **Location**	East	106 (50%)	4 (1.9%)	0.0006
	South	39 (18.4%)	10 (4.7%)	
	West	67 (31.6%)	9 (4.2%)	
**Place of Birth**	Khartoum State	193 (91%)	21 (9.9%)	1.0
	Outside Khartoum	19 (9%)	2 (0.9%)	
**Living outside Khartoum** **(first 2 yrs)**	No	193 (91%)	21 (9.9%)	1.0
	Yes	19 (9%)	2 (0.9%)	
**Father's Education Level**	Illiterate or elementary	65 (30.8%)	10 (4.7%)	0.1631
	Above elementary	146 (69.2%)	13 (6.2%)	
**Mother′s Education Level**	Illiterate or elementary	73 (34.6%)	13 (6.2%)	0.0192
	Above elementary	138 (65.4%)	10 (4.7%)	
**Feeding History**	Exclusive Breastfeeding	180 (85.3%)	20 (9.5%)	1.0
	Bottle Feeding	2 (1%)	0	
	Both	29 (13.7%)	3 (1.4%)	
**Duration of Breastfeeding**	< 6 months	6 (2.8%)	0	0.5572
	6–12 months	25 (11.9%)	4 (1.9%)	
	>12 months	180 (85.3%)	19 (9.0%)	
**Age (years)**	**Mean (SD)**	**Median (IQR)**		**Range**
	4.68 (0.88)	5 (4, 5)		3 – 8

* P < 0.05 were considered statistically significant (Chi-Square or Fisher's Exact Tests)

### Clinical IOM vs. history of IOM

Of the total study population, a positive history of IOM was reported in 11.8%, (n=25) of cases compared to 10.8% (n=23) with confirmed “clinical IOM”. McNemar's Test was used to compare “clinical IOM” and “history of IOM” and no significant differences between the marginal probabilities were found (p-value= 0.6698) (Table 2).

**Table 2:** McNemar′s Test to compare Clinical IOM and History of IOM

History of IOM	Clinical IOM		
	No	Yes	Total
**No**	177	10	187
**Yes**	12	13	25
**Total**	189	23	212

### Socio-demographics and teething-related risk factors of IOM

Geographical location (p-value=0.0006), mother's education level (p-value=0.0192), teething problems (p-value= 0.0445), diarrhoea (p-value=<0.0001) and vomiting (p-value=0.0005) were the statistically significant risk factors for clinical IOM (Table 3).

**Table 3:** Teething problems (n,%) of the sample population (n=212) and correlation with clinical IOM

Variable		N (%)	Clinical IOM	P-value*
Teething Problems	Yes	134 (63.5%)	20 (9.5%)	0.0445
	No	71 (33.7%)	3 (1.4%)	
	Don′t Know	6 (2.8%)	0	
Fever	Yes	78 (36.8%)	11 (5.2%)	0.2452
Gum Pain	Yes	19 (9.0%)	3 (1.4%)	0.4414
Drooling	Yes	14 (6.6%)	3 (1.4%)	0.1830
Biting	Yes	22 (10.4%)	3 (1.4%)	0.7147
Diarrhea	Yes	61 (28.8%)	16 (7.6%)	<0.0001
Vomiting	Yes	49 (23.1%)	12 (5.7%)	0.0005
Other teething symptoms	Yes	30 (14.2%)	2 (0.9%)	0.5431
Parents′ action to teething problems	Nothing	96 (45.3%)		
	Doctor	77 (36.3%)		
	Traditional Healer	28 (13.2%)		
	Pain Medication	11 (5.2%)		

* P < 0.05 were considered statistically significant (Chi-Square or Fisher′s Exact Tests)

### Dental findings

The resulting multivariable logistic regression analysis model contained two variables: Diarrhea and mothers' education. The strongest predictor of clinical IOM was diarrhea. Respondents who reported diarrhea were over seven times as likely to have clinical IOM than those who did not have diarrhea and mothers who were less educated (illiterate or had elementary school education) were more than twice likely to have children with clinical signs of IOM (Table 4).

**Table 4:** Multivariate logistic regression model for the predictors of IOM. [Odds ratios (ORs) with 95% confidence intervals (CIs) and P-value]

Variable	Overall Test p-value	OR (95% CI)
**Mother′s Education Level**		
Illiterate or Primary School vs. Above Primary School	0.0369	2.69 (1.06, 6.81)
**Diarrhea**	<0.0001	7.15 (2.73,18.69)

**Figure 1:**
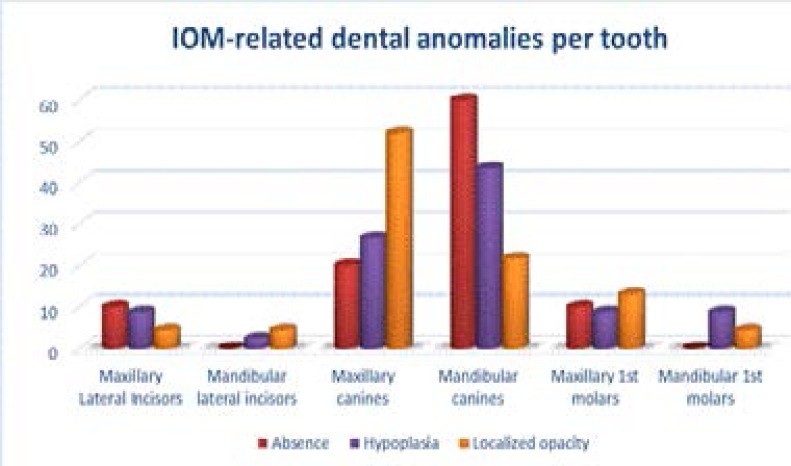
frequency (%) distribution of dental anomalies per tooth.

A total of 2544 teeth were examined. Seventy-nine teeth (3.1 %) showed dental anomalies associated with the practice of IOM. Enamel hypoplasia was the most common dental anomaly, accounting for 58.23% (n=46) of the affected teeth. Localised enamel opacities accounted for 29% (n= 23), while only 12.6 % were completely absent (n=10). In general, the tooth most affected by the IOM was the mandibular canine, representing about three-quarters of all affected teeth i.e 73.4 % (n=58). The mandible and the maxilla were almost equally affected by IOM. The maxilla was affected in 51.9% of cases whereas, in the mandible, 48.1 % were affected. The complete absence of canines was, however, more noticeable in the mandible compared with the maxilla (60 % vs. 40 % respectively). Figure 1 refers to distribution of IOM-related dental anomalies per tooth.

## Discussion

Based on evidence from observations in clinical practice in pediatric dental clinics in Khartoum, children with manifestations suggestive of the practice of IOM were seen on a frequent basis. This, together with the considerable number of recent case reports confirming IOM practice among Sudanese immigrant populations^15^,^16^ highlighted the need to update the prevalence data of IOM from Khartoum. To the best of our knowledge, there is only an early single published study documenting the practice of IOM in Khartoum^9^. The study sample was limited to government pre-schools in one district out of the seven districts within the Khartoum state thus capturing a particular geographical location. Yet, subjects were recruited through a random systematic sampling strategy within the pre-school strata warranting representation of the population.

### Statement of principal findings

The key finding of this study indicates that nearly one in each^10^ children in the inner-city child population we examined have confirmed clinical IOM. Comparison of this finding with the earlier IOM report from Khartoum^9^, which reported a higher IOM prevalence of 22%, may have its limitations since the earlier study employed different examination criteria and provided little description of the geographical area of the study location within the city of Khartoum . Furthermore, more cases of “Lugbara tooth” referring to complete enucleation of the primary canine tooth germs were found in the present study (ten teeth were completely enucleated out of the total 79 teeth) compared to only one case in the study by Rasmussen^9^. This may reflect an aggressive shift in the practice of IOM by traditional healers in Khartoum, contradicting the assumption that the practice is mainly limited to lancing the gingiva. Yet, the legitimacy of comparisons remain inadequate since our data were geographically limited to a single district and socio-economic group, therefore necessitating the extension of comparable surveys to include different population demographics within and outside Khartoum state.

It is of considerable importance to point out that our study reported two different prevalence figures based on either the “clinical IOM” or the “parental confirmation” of traditional healer visit. Prevalence figures for “clinical IOM” drop to six percent (one child in every 16 children) if we take into account clinical IOM cases with a positive parental report. Our study revealed that fewer parents would report tooth enucleation than the actual evident clinical IOM. Although this difference was not statistically significant, we needed to identify the amount of existing discrepancies since the report of a traditional healer visit by the parent/guardian is vulnerable to negation rather than confirmation and relies exclusively on the parental memory thus susceptible to possible recall bias. In this study, subjects were believed to constitute a homogeneous group based on the fact that government preschools incorporate children mostly from low or very low socio-economic class families in the city of Khartoum. More than ninety percent of the subjects included in the study were inner city children who were born and resided in Khartoum for the first two years. Interestingly, a very negligible percent (less than 1%) of those children who showed clinical evidence of IOM were born and resided outside Khartoum, confirming our assumption that this ritual is established among inner city children.

The second key finding of our study outlines that some socio-demographic risk factors such as the residence geographical location and mother's education level are strongly correlated to IOM in this child population. Authors are aware that the significant findings related to these specific findings need to be interpreted with caution because of the skewed distribution of data towards more mothers with education above elementary level and more subjects residing in the East side of Khartoum district. IOM practice has long believed to be exclusively prevalent in rural communities^5^,^7^,^8^. However, our report provides some proof that IOM is widespread as well among inner city children as the majority of our study population was assumed inner city residents of the city of Khartoum. Furthermore, there were some geographical trends in the distribution of IOM cases. We were not able to provide any explanation of why children who resided in the South of the city of Khartoum were significantly at risk of showing clinical IOM. The authors believe that racial/ethnic concentrations within certain geographical locations within the city can be quoted as another IOMrelated issue^21^,^22^. The racial/ethnic aspects as risk determinant of IOM were, however, not explored in the present study because of their apparent sensitivity even though this specific geographical location of the city (Soba area) is a known ‘low-income high density area’ incorporating one of the largest slum areas of internally displaced groups in Khartoum district^23^. The conclusion that there are geographic-related IOM trends coincides with results of a previous Ugandan study where intra-country variations of IOM among the different districts existed^22^.

On the other hand, our results revealed that mothers'^9^,^18^ or head of the household's education^20^ is strongly correlated with the manifestations of IOM which is in agreement with previous reports. Unquestionably, educated mothers were found to be more aware of their children health issues, more prepared to deal correctly with the problems^24^, and less likely to adopt the practice of IOM^21^. In accordance with a recent report^21^, our data showed that the strongest IOM predictor of teething-related symptoms was childhood diarrhea. Comparable to the findings from a study investigating the attitude and practice of mothers regarding diarrhea in infants in a Sudanese rural community^25^, almost one-third of our targeted inner city parents who recalled diarrhea as a frequent teething symptom opted for removing a child's tooth buds as an effective treatment for childhood diarrhea. Despite the possible methodological limitations related to lack of precision of parents reporting teething symptoms, history of childhood diarrhea seems to be a significant predictor in the identification of children who are at risk of IOM. Regarding the clinical distribution of dental anomalies, mandibular canines were the most commonly affected teeth, which is consistent with previous studies^6^,^9^,^18^. This can be attributed to the fact that the marked bulging of the mandibular canine makes them easily visible and accessible for enucleation^6^.

### Limitations of the study

The authors are aware that underlying risk factors of IOM are highly complex and multifaceted, and it is likely that several factors such as social and cultural components of the practice have an impact on the prevalence figures of IOM. Children from low socio-economic families have limited access to primary health care services and therefore overburdened with infectious diseases^20^. Limiting the study population to one socio-economic group may have biased our prevalence estimates of IOM. However, our data possibly paves the way for further studies examining children from all different socio-economic backgrounds.

### Unanswered questions and future research

The evidence provided by this report fosters the demand for not only additional quantitative reports from other districts in Khartoum, but for qualitative studies exploring the logical, cultural, social, and economical motives of adopting such a deleterious practice from the mothers, guardians, and traditional healers' perspectives. Furthermore, comparative research studies from different African inner-city populations might probably unlock many queries about the cultural and socio-economic determinants of this practice.

## Conclusion

Infant oral mutilation (IOM) is a common practice among inner city children in Khartoum. Our quantitative data demonstrated that certain teething-related symptoms especially diarrhea and maternal education background could be strong determinants of the malpractice of IOM.

## Declarations

### Availability of data and material

The datasets used and/or analysed during the current study were available from the corresponding author on reasonable request.
